# An integrative study identifies *KCNC2* as a novel predisposing factor for childhood obesity and the risk of diabetes in the Korean population

**DOI:** 10.1038/srep33043

**Published:** 2016-09-14

**Authors:** Joo-Yeon Hwang, Hyo Jung Lee, Min Jin Go, Han Byul Jang, Sang Ick Park, Bong-Jo Kim, Hye-Ja Lee

**Affiliations:** 1 Center for Genome Science, National Institute of Health, Osong Health Technology Administration Complex, Chungcheongbuk-do, Republic of Korea; 2 Center for Biomedical Sciences, National Institute of Health, Osong Health Technology Administration Complex, Chungcheongbuk-do, Republic of Korea

## Abstract

Obesity is a major risk factor for type 2 diabetes. To unravel the genetic determinants of obesity-associated diabetes, we performed a genome-wide study using the 1,000 Genomes-based imputation in a Korean childhood cohort (KoCAS-1, n = 484) and carried out *de novo* replication in an independent population (KoCAS-2, n = 1,548). A novel variant (rs10879834) with multiple diverse associations for obesity-related traits was also found to be replicated in an adult cohort (KARE, n = 8,842). Functional annotations using integrative epigenetic analyses identified biological significance and regulatory effects with an inverse methylation-expression correlation (cg27154343 in the 5′-UTR of the *KCNC2* gene), tissue-specific enhancer mark (H3K4me1), and pathway enrichment (insulin signaling). Further functional studies in cellular and mouse models demonstrated that *KCNC2* is associated with anti-obesogenic effects in the regulation of obesity-induced insulin resistance. *KCNC2* shRNA transfection induced endoplasmic reticulum (ER) stress and hepatic gluconeogenesis. Overproduction of *KCNC2* decreased ER stress, and treatment with metformin enhanced *KCNC2* expression. Taken together, these data suggest that reduction of *KCNC2* is associated with modified hepatic gluconeogenesis and increased ER stress on obesity-mediated diabetic risk. An integrative multi-omics analysis might reveal new functional and clinical implications related to the control of energy and metabolic homeostasis in humans.

Obesity is a serious medical disorder characterized by hyperplasia (cell number increase) and hypertrophy (cell size increase)^1^. The worldwide epidemic of obese and overweight children has been increasing in Westernized and industrialized countries^2^. Higher BMI during childhood leads to obesity in adulthood with serious health consequences such as high blood pressure, type 2 diabetes (T2D), hyperlipidemia and related metabolic disorders^3^,^4^,^5^.

Considering the prevalence and heritability of childhood adiposity, genetic variations^6^ might play an important role in the lifelong effects of childhood obesity under obesogenic environments^7^,^8^. To date, genome-wide associations related to body mass index (BMI) or obesity-related traits have been predominantly found in cohort-based European-ancestry populations^9^,^10^,^11^,^12^, within only limited data from Asian population^13^,^14^. Two studies have recently reported new common variants underlying childhood obesity in Europeans^15^,^16^. However, the functional and biological significance are still not fully understood in the polygenic architecture of common complex diseases.

Recent epigenome-wide association studies (EWAS) have identified new susceptibility loci with specific epigenetic modifications and genomic features^17^,^18^. As a part of an integrated functional genomics strategy, epigenetic variations can contribute to the missing heritability in T2D and related metabolic traits^19^. In this study, we performed genetic-epigenetic association analyses for childhood obesity and the risk of T2D in the Korean population. Also, we systematically investigated its functional effects on trait-determining cell types and mouse models.

## Results

**Figure 1:**
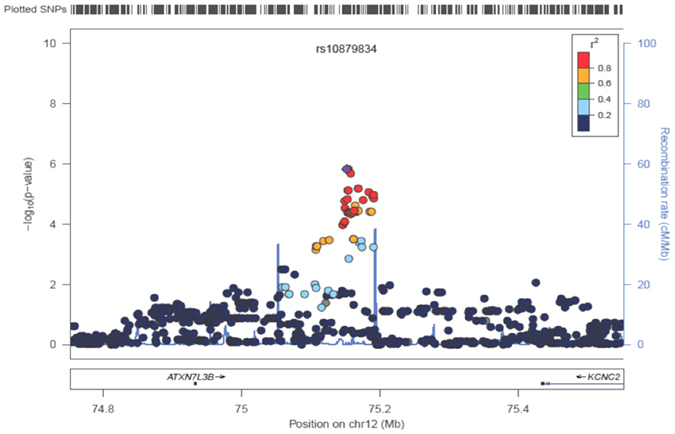
A regional association plot for the rs10879834 SNP.

To identify novel genetic susceptibility loci for childhood obesity, we conducted a genome-wide association study (GWAS) screen with the 1,000 Genomes-imputed data in a Korean childhood cohort (n = 484). Genotyping for *de novo* replication was carried out in an independent pediatric population (n = 1,548). The clinical characteristics and statistics for each study sample are described in Table 1. Following standard quality control criteria, all 357,789 SNPs were tested using multiple linear regression analysis after statistical adjustment for age and sex. The Manhattan plot showing the genome-wide results for the genotyped and imputed datasets is presented in Supplementary Figure 1. The genomic control inflation factor (λ) was 1.007 for the assessment of population structure. The Q-Q plot for the trend test showed that the distribution of observed *p*-values deviated from expected *p*-values only in the extreme tail, suggesting true-positive association signals (Supplementary Figure 2). In the discovery GWAS analysis, no genetic variants reached the conventional genome-wide significance threshold (*P* = 5 × 10^−8^). However, three SNPs harboring the *ATXN7L3B*-*KCNC2* (intergenic, rs10879834), *NTM* (intronic, rs2512887), and *SOX5* (intronic, rs10505912) genes were newly found to be associated with BMI (KoCAS-1, n = 484) (Supplementary Table 1). Of these, SNP rs10879834 in *KCNC2* was significantly replicated in an independent childhood cohort (KoCAS-2, n = 1,548) with the same direction of association. We also observed multiple diverse effects with significant associations in obesity-related traits (waist, weight and hip). Additionally, SNP rs10879834 showed effects that were directionally consistent with those of the adult BMI and obesity-related traits (KARE, n = 8,842) (Table 2 and Fig. 1). Moreover, the variant rs10879834 showed the significant association with fasting plasma glucose in KoCAS-1 population. It showed similar effect in KoCAS-2 but not significant.

**Figure 2:**
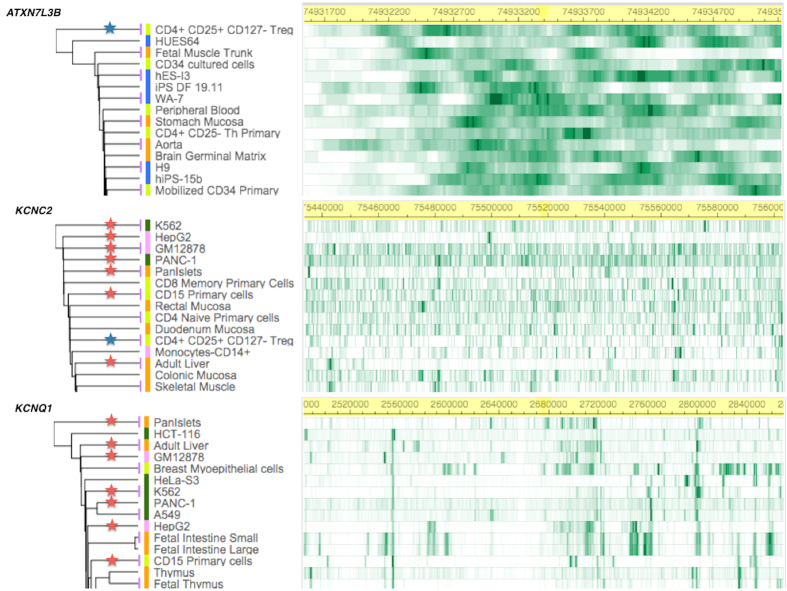
Epigenetic functional annotations using tissue-specific hierarchical clustering. Based on the top-down approach, hierarchical clustering is applied to H3K4me1 (mark of regulatory elements associated with enhancers) using primary cells (light green), primary tissues (orange), and cell lines (blue), and cancer cells (green). H3K4me1 chromatin immunoprecipitation (ChIP)-seq read density (in green) is shown for each gene. A blue star indicates overlapping enrichment between *ATXN7L3B* and *KCNC2*. Red stars (n = 7) indicate overlapping enrichment between *KCNC2* and *KCNQ1*.

**Figure 3:**
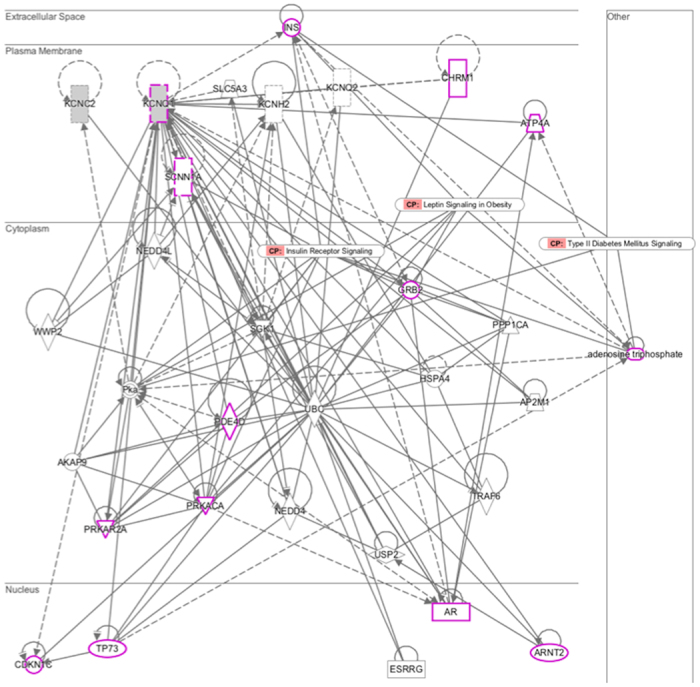
Sub-cellular network identified by means of the Ingenuity Pathway Analysis (IPA). All factors are based on molecular transport in IPA (*p* = 2.44e-09). Pink indicates previously established T2D-factors for three canonical pathways (i.e. insulin receptor signaling, leptin signaling in obesity, and type II diabetes mellitus signaling). Solid and dashed lines indicate direct and indirect interactions, respectively. The arrows indicate specific molecular relationships. Nodes are displayed by the functional classes of the gene product (square, cytokines; diamond, enzyme; circle in a circle, complex/group; triangle, kinase; rectangle, ligand-dependent nuclear receptor; dotted rectangle, ion channel; trapezium, transporter).

**Figure 4:**
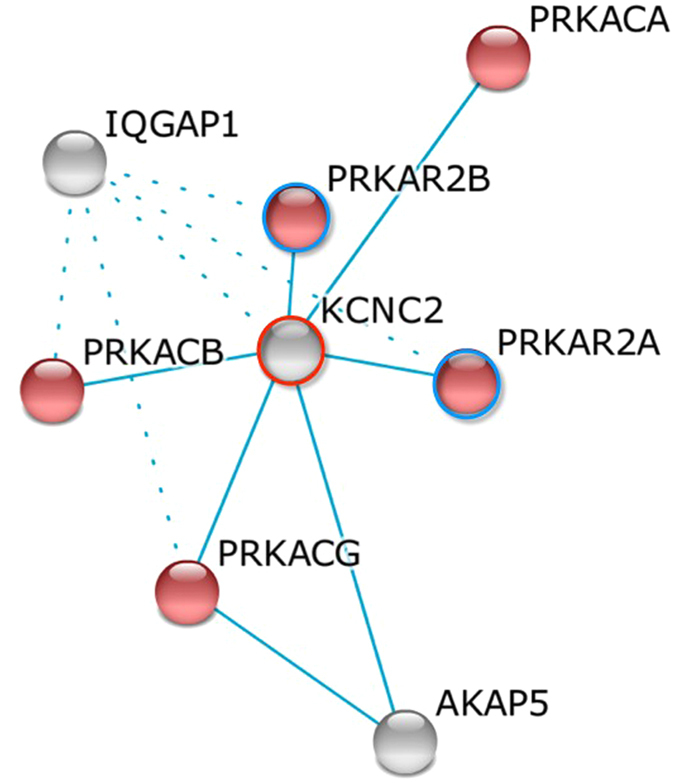
An integrative functional network in an insulin-signaling pathway. A gene network by k-mean clustering was constructed with insulin signaling pathway enrichment (red color, *p* = 8.2e-8). Filled colors indicate the genes up-regulated (blue circles) and down-regulated (red circles) by GEO expression analyses.

To evaluate the functional significance of the newly discovered intergenic variant (rs10879834), we analyzed epigenetic regulatory effects using the Roadmap Epigenome Browser, clustering the histone H3K4me1 profile of SNP-harboring regions between *ATXN7L3B* and *KCNC2*. Compared with the common enrichment of enhancer marks in *ATXN7L3B* across cell types, *KCNC2* was found to be associated with enhancer-specific marks in physiologically or pathogenically relevant tissue cell types such as the pancreatic islet and adult liver. We also observed a consistent stratification of enrichment in *KCNQ1* as a previously established T2D gene^20^ (Fig. 2). To compare common biological associations between *KCNC2* and *KCNQ1*, we performed pathway analysis using the Ingenuity Pathway Analysis (IPA). Sub-cellular network analysis demonstrated the shared biological significance underlying three canonical signaling pathways: insulin receptor, leptin signaling in obesity, and T2D (Fig. 3). To predict functional effects for genotype-methylation correlations, we first tested *cis-me*QTL analysis using the MuTHER resource (adipose tissue collected from 856 healthy female twins). We identified a significant DNA methylation site (CpG ID: cg27154343) in the *KCNC2* gene (Supplementary Figure 3). Given epigenetic heterogeneity between ethnic groups, we performed a replication study in a Korean population. Using our T2D-discordant monozygotic twin model (n = 12 pairs) (Supplementary Table 2), we were also able to validate a significant epigenetic association of T2D. The CpG site (cg27154343) in the *KCNC2* was found to be significantly associated with T2D risk as a differentially methylated region (DMR) (Table 3). To examine the correlation between human gene expression and protein-protein interaction (PPI), we conducted gene expression profiling analyses using the public GEO database resource (GSE26168). We found that *KCNC2* mRNA expression levels were significantly down-regulated in T2D cases (n = 9) compared with controls (n = 7) (*P* = 3.46 × 10^−2^) (Supplementary Figure 4). The *KCNC2*-centered protein-protein networks were constructed with the strongest significant connecting terms on apoptosis- and insulin signaling-pathway enrichment (*P* = 1.65 × 10^−8^ and *P* = 8.20 × 10^−8^) against KEGG pathway maps. An integrative functional connectivity based on GEO and PPI was highlighted in Fig. 4.

**Figure 5:**
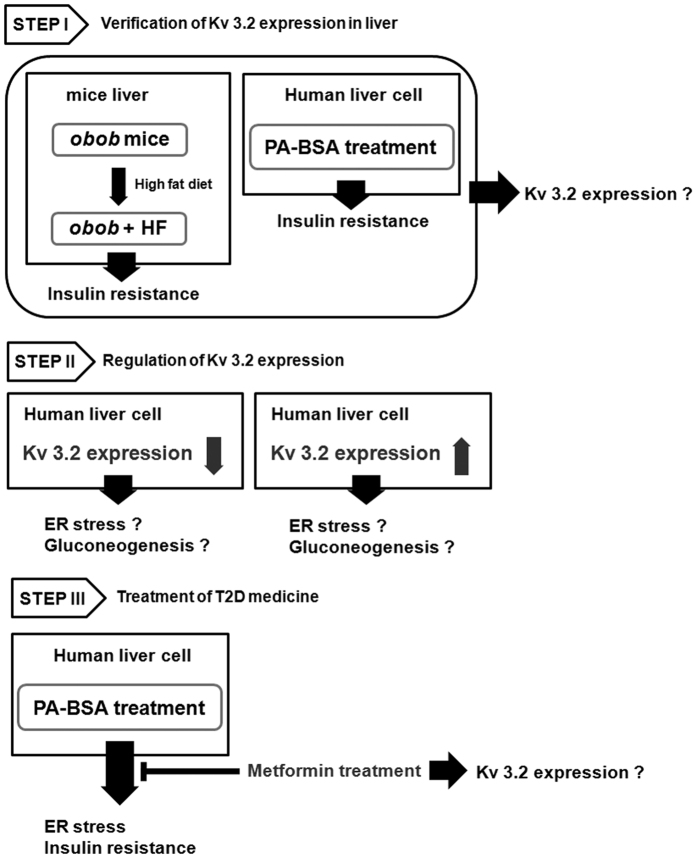
The flow chart for experimental model. Step I: Leptin-deficient (*ob/ob*) mice were characterized by type 2 diabetes with mild hyperglycemia and obesity. Feeding of high-fat diet or excess palmitate induces not only obesity but also insulin resistance. We investigated Kv 3.2 expression in obesity-induced insulin resistance. Step II: We identified whether Kv 3.2 expression regulates insulin resistance including ER stress and gluconeogenesis or not. Step III: Metformin (1, 1-dimethylbiguanide hydrochlolride) is a biaguanide commonly used to treat type 2 diabetes mellitus. Metformin attenuates the response of ER stress which is induced by excess free fatty acid. Also, metformin increases insulin sensitivity through the decrease of serine phosphorylation of IRS-1 which is increased by palmitate-induced ER stress. Therefore, we confirmed whether metformin modulates Kv 3.2 expression or not.

**Figure 6:**
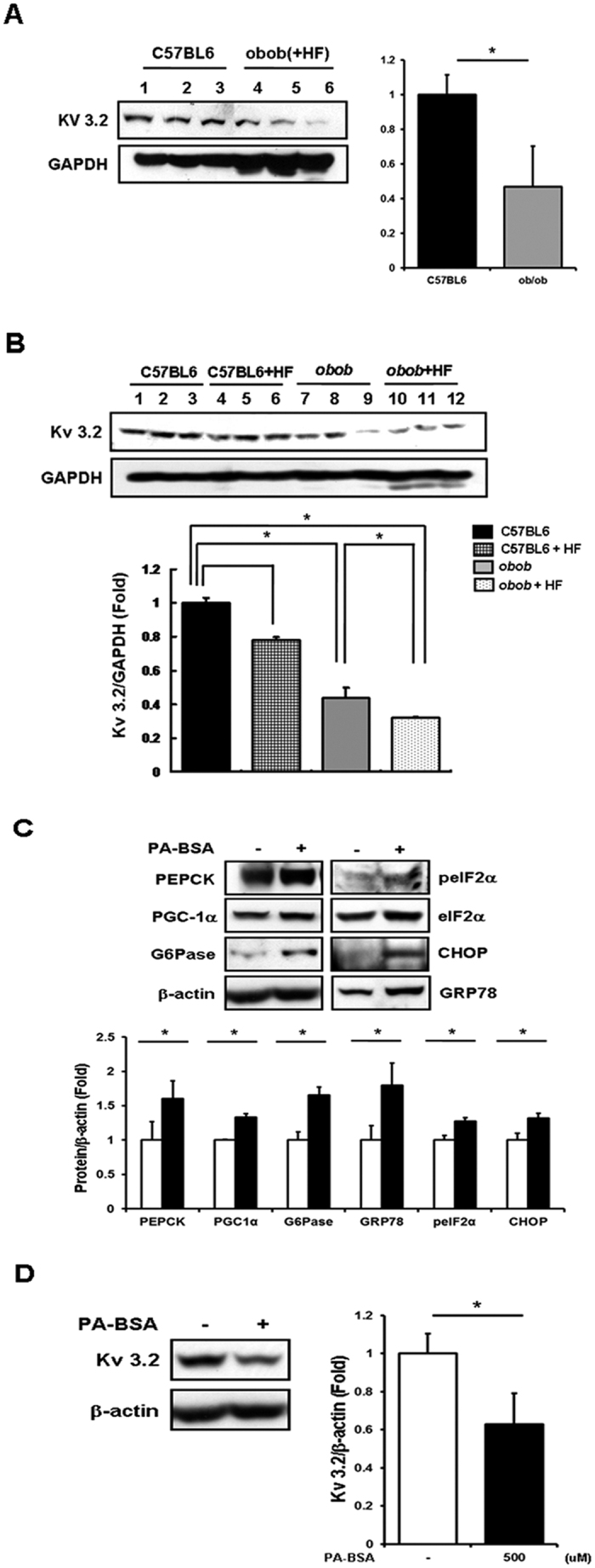
Change of hepatic Kv 3.2 expression in obese mice and palmitate treated liver cells. (**A,B**) Whole lysate was extracted from liver tissue of 7 week-old male *ob/ob* with/without high-fat diet for 3 weeks and age-matched lean mice; western blotting was performed. (**C**) Sk-Hep I cells treated with palmitate (500 uM) for 24 h. The expression levels of protein and transcription factors related to gluconeogenesis and the ER stress response were analyzed in whole cell lysate by western blotting. (**D**) Kv 3.2 expression was analyzed in whole cell lysate by western blotting. *Presents significant differences between groups at *p* < 0.05. All data were represented as means ± SD, n = 3. Middle-length blots and two exposures are presented in Supplementary Figure 5.

**Figure 7:**
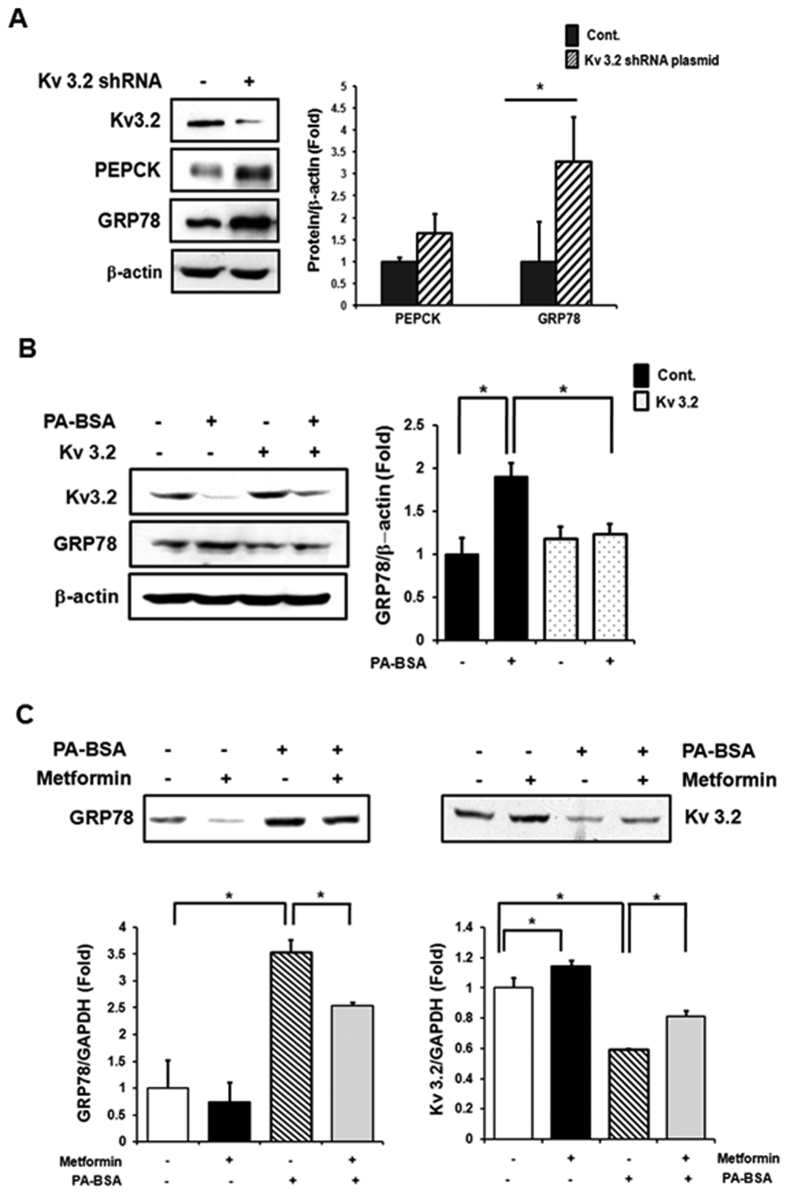
Kv 3.2 is associated with gluconeogenesis and ER stress response, and Kv 3.2 expression is increased by metformin. (**A**) Sk-HepI cells infected with Kv 3.2 shRNA plasmid for 48 h, followed by western blotting. (**B**) Sk-HepI cells infected with Kv 3.2-pCMV6 for 48 h and then treated with palmitate (24 h, 500 uM), followed by western blotting. (**C**) Sk-HepI cells treated with palmitate (500 uM) without metformin (3 mM) for 24 h. Sk-HepI cells were pre-treated with Metformin for 2 h before palmitate treatment, followed by western blotting. *Presents significant differences between groups at *p* < 0.05. All data were represented as means ± SD, n = 3. Middle-length blots and two exposures are presented in Supplementary Figure 6.

To expand the functional implications of *KCNC2,* we studied its biological interactions using disease-specific cellular and mouse models and explained the experimental model with flow chart in Fig. 5. We first studied protein expression levels in liver tissues of *ob*/*ob* mice and age-matched lean mice (C57BL/6J). The leptin-deficient *ob*/*ob* mice, characterized by hyperphagia-induced insulin resistance, are a well-established model for obesity^21^. Hepatic Kv 3.2 expression was significantly down-regulated in *ob*/*ob* mice (Fig. 6A, Supplementary Figure 5A). In particular, we observed reduced expression of *KCNC2* in *ob*/*ob* mice fed a high-fat diet (Fig. 6B, Supplementary Figure 5B). To investigate the metabolic profile in a human-derived liver cell line, we measured hepatic enzyme expression levels in palmitate-treated SK-Hep I cells. There were significant changes in increased expression levels of 3 gluconeogenesis-(PEPCK, PGF-1α and G6Pase) and 4 ER stress-related factors (peIF2α, eIF2α, CHOP and GRP78) (Fig. 6C, Supplementary Figure 5C), respectively. We also found that Kv 3.2 expression was significantly down-regulated in palmitate-induced gluconeogenesis and the ER stress response (Fig. 6D, Supplementary Figure 5D). shRNA transfection revealed that absence of Kv 3.2 is associated with increased expression levels of PEPCK and GRP78 (Fig. 7A, Supplementary Figure 6A). Moreover, we confirmed that overexpression of Kv 3.2 alleviates palmitate-induced ER stress via down-regulation of GRP78 expression (Fig. 7B, Supplementary Figure 6B), but we found no consistent effects on gluconeogenesis (data not shown). To understand the reciprocal regulation underlying abnormal gluconeogenesis and the ER stress response in T2D, we confirmed the increase of Kv 3.2 expression in metformin treated cells (Fig. 7C, Supplementary Figure 6C). These findings demonstrate that Kv 3.2 is functionally associated with anti-obesogenic effects on hepatic gluconeogenesis and the ER stress response.

## Discussion

Childhood obesity has been recognized as a serious global health problem with a high prevalence^22^. Childhood obesity carries an increased risk of adult obesity. Higher BMI during childhood leads to adult obesity and metabolic diseases such as T2D^3^,^4^,^23^. However, to date, genetic susceptibility loci have been identified primarily in adult cohort-based samples of European descent. Few GWAS studies have reported genetic loci conferring a predisposition to childhood obesity in populations with European-ancestry^6^,^15^,^16^.

To attain higher genomic coverage and resolution, we conducted a genome-wide association study using the 1,000 Genomes-based imputation data for the purpose of discovering new loci influencing childhood obesity. Our study identified a novel *KCNC2* gene as a predisposing factor for susceptibility to both childhood- and adult- obesity in the Korean population. However, we did not obtain genome-wide significant support for associations with early-onset obesity, although a newly identified SNP rs10879834 showed directionally consistent associations with pleiotropic effects on obesity-related traits.

Within 500 kb flanking regions containing several pseudo genes, the only RefSeq genes are *ATXN7L3B* and *KCNC2*, which are approximately 220 kb proximal to the GWAS signal (rs10879834). A structural variation study highlighted that a new chromosomal deletion including two genes (*ATXN7L3B* and *KCNC2*) is associated with human neurodevelopmental delay^24^. The genes within 2 Mb of the signal, include *RP11-81H3.2*, *CAPS2*, *GLIPR1*, *GLIPR1L1*, *GRLPR1L2*, and *KRR1*, none of which is an obvious candidate gene for involvement in obesity or T2D. Recent studies reported that genetic variants in potassium channels are associated with insulin response in child^25^ as well as in adult populations^26^. Additionally, functional studies have demonstrated that inhibition of the voltage-gated potassium channel Kv 1.3 has a strong anti-obesogenic effect in mice^27^,^28^,^29^. It has been suggested that alterations of potassium channels may also counteract obesity-triggered vascular dysfunction^30^,^31^.

Potassium channels are classified into three main families based on their transmembrane structure. Different subtypes encoded by 75 genes are involved in a variety of physiological and pathological functions^32^. Kv 3.2 is a member of the Kv 3 channel subfamily. Kv 3.2 channel (*KCNC2*, potassium voltage-gated Shaw related subfamily C) is prominently expressed in fast-spiking GABAergic interneurons^33^,^34^. It has previously been established that leptin’s anti-obesity effects are mediated by leptin-responsive GABAergic neurons^35^. Double knock-out mice (lacking *KCNC1* and *KCNC2* genes) exhibited the impacts of physiological properties on the circadian oscillations^36^, suggesting that Kv 3 channels may serve as a potent target for studying the roles of these channels in the regulation of energy and metabolic homeostasis. It has long been recognized that circadian disruption is critical to metabolic abnormalities, including central obesity, fasting glucose level, diabetes and hypertension^37^. Altogether, the evidence suggests that Kv 3.2 channels may serve as a potent candidate for studying these channels and for understanding the functional regulation of obesity-associated T2D risk.

Despite the well-characterized functional implications of the voltage-dependent potassium channels, nothing is known about the role of these Kv 3.2 channels in obesity-associated metabolic status. To develop rational hypotheses for the functional roles uncovered by GWAS, we analyzed epigenomic regulatory features to implicate particular genes and pathways for functional annotations. Hierarchical clustering analyses using the Roadmap Epigenome Browser support that cell-type specific enhancer clusters provide important clues for the functional relevance of *KCNC2* as a candidate gene^38^. We also confirmed consistent enrichment between the *KCNC2* and *KCNQ1* underlying canonical pathways including insulin receptor, letpin, and T2D signaling pathways, using IPA analysis^39^. In *cis-me*QTL analyses using adipose tissue data from a population of 428 female twins from the MuTHER data, we found novel evidence suggesting that DNA methylation status in the *KCNC2* may provide promising data on the functional consequences of the effects of the observed genetic associations on obesity^40^,^41^. DNA methylation has an important potential role in improving etiological understanding related to T2D development. Recently, an integrated epigenomic analysis for T2D in monozygotic twins demonstrated that ~70% of the observed DMRs at T2D-GWAS loci are hypermethylated. Of these, *KCNQ1*, *KCNJ11* and *KCNK16* are hypomethylated in the T2D-DMR genome-wide distribution^20^. In this study, we observed a significant DMR association of *KCNC2* using genome-wide DNA methylome profiles in our T2D-discordant monozygotic twin model. This result was supported by gene expression profiling analyses using the GEO database. We found that *KCNC2* expression values were inversely correlated with DNA methylation levels at a CpG located in the 5′-UTR region. Negative relationships between gene expression and promoter DNA methylation have been previously reported in gene regulation via epigenetic changes^42^,^43^. These findings highlighted the biological functions of the newly identified *KCNC2* gene in the regulation of obesity-associated T2D risk.

Finally, there were consistent functional contributions in experimental cellular and mouse models. Using *in vivo* analysis, we observed that hepatic expression levels of Kv 3.2 were significantly down-regulated in *ob*/*ob* mice fed a high-fat diet as well as in leptin-deficient (*ob/ob*) mice. Using *in vitro* analysis, the palmitate-induced cellular insulin resistance underlying gluconeogenesis and ER stress was confirmed by the reduced Kv 3.2, ER stress signal mediator, and gluconeogenic enzyme gene expression. Excessive plasma free fatty acids are known to associate with obesity-mediated insulin resistance^44^,^45^,^46^,^47^. A systematic analysis of gene knockdown and overexpression, found that Kv 3.2 was significantly associated with anti-obesogenic effects. GRP78 expression, which is a major ER chaperone in the initiation stage under stress conditions, was increased by absence of Kv 3.2. Interestingly, elevated- GRP78 expression due to excessive cellular palmitate was improved by Kv 3.2 overexpression. However, there was no consistent change in PEPCK expression as a function of overexpression of Kv 3.2, whereas PEPCK expression was increased by Kv 3.2 knockdown. Under stress conditions, induced ER stress can contribute to the development of insulin resistance, which leads to abnormal gluconeogenesis. Therefore, Kv 3.2 may directly affect the ER stress response but not gluconeogenesis. We further observed that metformin treatment leads to significant changes in *KCNC2* gene expression in normal SK-Hep I human liver cells. Metformin is an anti-diabetic drug for the treatment of T2D, particularly, in overweight and obese individuals. It works mainly by suppressing excessive hepatic glucose production through a reduction in gluconeogenesis^48^,^49^. Additionally, metformin improved insulin sensitivity through the decrease in the serine phosphorylation of IRS-1 that increased palmitate-induced ER stress^50^,^51^. We identified that Kv3.2 expression was enhanced by metformin in Fig. 7. These results indicate that Kv 3.2 expression is associated with obesity-mediated diabetes.

In conclusion, our integrative analyses of genetic variation and epigenetic regulation identified a novel *KCNC2* as a predisposing factor in conferring susceptibility to obesity-associated T2D risk in the Korean population. Follow-up functional studies demonstrated the anti-obesogenic effects of Kv 3.2 using well-established cellular and mouse models. Our results suggest that Kv 3.2 channels may participate in the pathways that regulate energy balance and metabolic homeostasis via a voltage-gated potassium channel. After further validation studies to determine genotype, epigenotype and phenotype dependencies, Kv 3.2 could be a new therapeutic agent for insulin resistance by inducing ER stress.

## Materials and Methods

### Study population

For KoCAS-1, 484 students, between the ages of 8 and 13 years, were recruited from Seoul and Kyunggi Province in 2010. KoCAS-2 1,548 students between the ages of 8- and 13- years were recruited from Seoul and Kyunggi province between 2006 and 2012. This study was performed as a part of the KoCAS, which has been monitoring a group of subjects annually since their entry into elementary school at age 7 years in 2005. Subjects enrolled in a specific diet program or subjects who were taking any medications known to affect appetite were excluded from the study. The study protocol was approved by the Institutional Review Board of Seoul-Paik Hospital, Inje University, (SIT-2010-052), and the Korea Center for Disease Control and Prevention (2012-04EXP-06-R). As indicated in the ethics statement, written informed consent was obtained from the children’s parents. The KARE study was conducted through the Korean Genome Epidemiologic Study project. Starting in 2001–2002, a total of 10,038 people between the ages of 40- and 69 years living in Ansung and Ansan were followed. The results of this study have been previously reported^52^,^53^. All study protocols were carried out in accordance with approved guidelines.

### Genotyping

A childhood obesity study was genotyped using the Illumina Omni1-Quad BeadChip. Individuals were excluded based on the following criteria: genotyping call rate, sex inconsistency, heterozygosity, identity-by-state (IBS) value and any type of tumor. The results of this study have been previously reported^52^. KARE samples were genotyped using Affymetrix Genome-Wide Human SNP array 5.0, and the Bayesian Robust Linear Modeling was processed using the Mahalanobis Distance (BRLMM) Genotyping Algorithm^54^.

### SNP imputation

Imputation of genotypes to the 1,000 Genomes phase I integrated variant call set release (version 3) in NCBI build 37 (hg19) as a reference panel was carried out using the IMPUTE (v2.644) (high imputation quality: proper info >0.5). Of these, we dropped SNPs with a posterior probability score <0.90, low genotype information content (info <0.5), HWE (*P* < 1 × 10^−7^), MAF <0.01, and SNP missing rate >0.1.

### Association analysis

SNPs were analyzed with the R (2.15.1) software package, PLINK (http://pngu.mgh.harvard.edu/~purcell/plink), and SAS programs (version 9.1; SAS Institute Inc., Cary, NC, USA). BMI and obesity-related traits were tested by multivariate linear regression analysis in an additive genetic model (1-d.f.) after adjustment for age and sex as covariates.

### Integrative functional analysis

To explore the tissue-specific regulatory roles of genetic variants, epigenomic annotations were visualized by clustering the histone enhancer mark (H3K4me1) profile of SNP-harboring regions using the Roadmap Epigenome Browser (http://epigenomegateway.wustl.edu/browser/roadmap/). The browser provides important clues into the functional relevance or epigenetic modifications across diverse human cell types to predict relationships between regulatory elements and target non-coding SNPs^38^. Interactive pathway analysis was performed using the Ingenuity Pathway Analysis (IPA) (http://www.ingenuity.com/products/ipa). Sub-cellular network analysis demonstrated a shared canonical pathway. The SNP- CpG associations were analyzed by incorporating methylation quantitative trait loci (*cis*-meQTL) information from the Multiple Tissue Human Expression Resource (MuTHER)^55^. The gene expression data set (GSE26168/GPL6883) was retrieved from the NCBI Gene Expression Omnibus (GEO). Functional connectivity and network were analyzed by the STRING database (http://string-db.org/)^56^. Interactions of the genes in the insulin signaling-related cluster were accessed using the KEGG pathway database resource. Differentially expressed genes with statistical significance were visualized as either up-regulated (red) or down-regulated (blue).

### Chemicals and animals

Palmitate and metformin were purchased from Sigma (St. Louis, MO, USA). Kv 3.2 shRNA plasmid and Kv 3.2 plasmid were obtained from Santa Cruz Biotechnology (Santa Cruz, CA, USA) and Origene Technologies (Rockville, MD, USA), respectively. Palmitate- BSA solution was prepared by dissolving palmitate in ethanol and then mixing it with fatty acid-free BSA (2% wt/vol. in water: Sigma) at 37 °C with shaking for 2 h. We obtained 7 week-old male *ob/ob* mice and age-matched lean mice (C57BL/6J) from the Animal Center of SLC, Inc. (Hamamatsu, Shizuoka, Japan); they were housed in individual cages at 22 ± 2 °C with a 12-h light-dark cycle. Subjects were divided into two groups: the first group received a standard chow diet (Purina #5001 Chow; Dyes Inc. Bethehem, PA, USA), and the second group received a high-fat diet (45% of energy from fat) for 3 weeks. After overnight fasting, the liver was removed from each mouse and used for western blot analysis. All animal experiments were approved by an appropriate institution/licensing committee, at the Korea National Institute of Health Animal Facility (KCDC-015-11-2A). The methods were carried out in accordance with the approved guidelines.

### Cell culture

The SK-Hep I human liver cell line (ATCC CRL 1772; ATCC, Manassas, VA, USA) was cultured using Dulbecco’s modified Eagle’s medium (DMEM) supplemented with 10% fetal bovine serum (FBS) and antibiotics. However, we cultured these cells using DMEM supplemented with 2% FBS and antibiotics when they were treated with PA-BSA solution (500 μM) for 24 h.

### Kv 3.2 transfection

Kv 3.2 shRNA plasmid (#62534-SH) and control shRNA plasmid (#108060) were purchased from Santa Cruz Biotechnology. Cells were transfected with 4 μg of shRNA plasmid per 1.5 × 10^5^ cells using Lipofectamine PlusTM reagent (Invitrogen) according to the manufacturer’s instructions. To confirm the change in Kv 3.2 (#RC222290) expression, total protein was extracted after 48 h of transfection and western blot analysis was performed using anti-Kv 3.2 antibody. In the case of stress induction, cells were treated with or without PA-FBS at 24 h after transfection and incubated for a further 24 h.

### Western blotting

At the end of each treatment, whole cell lysate was prepared by incubation on ice with lysis buffer (50 mM Tris-Cl (pH 7.5), 20 mM NaCl, 5 mM EDTA, 1% TX-100, 0.1% SDS, 5% glycerol and protease inhibitor), followed by ultrasonication for 10 s (Sonics & Materials Inc., Newtown, CT, USA). After centrifugation at 12,000 rpm for 20 min, the supernatants were subjected to SDS-PAGE and then transferred to a PVDF membrane. After transfer, the membrane was blocked and then probed with antibodies. Immunoblots were visualized using a ECL chemiluminescence detection kit (Thermo Scientific, Meridian Rd, USA). Anti-Kv 3.2 antibody was purchased from Sigma and Santa Cruz Biotechnology. Antibodies against eukaryotic translation initiation factor 2α (eIF2α) and peIF2 were acquired from Cell Signaling Technology (Beverly, MA, USA). All other antibodies were purchased from Santa Cruz Biotechnology.

## Additional Information

**How to cite this article**: Hwang, J.-Y. *et al*. An integrative study identifies *KCNC2* as a novel predisposing factor for childhood obesity and the risk of diabetes in the Korean population. *Sci. Rep.* **6**, 33043; doi: 10.1038/srep33043 (2016).

## Supplementary Material

Supplementary Information

## Figures and Tables

**Table 1:** Summary of study population characteristics.

Study	KoCAS-1	KoCAS-2	KARE
Sample size	484	1548	8842
Age (yr)	10.83 ± 1.43	10.60 ± 1.36	52.22 ± 8.92
Male/Female	232/252	750/798	4183/4689
BMI (kg/m2)	18.97 ± 3.82	18.43 ± 3.04	24.60 ± 3.12
BMI z-score	0.16 ± 1.03	−0.02 ± 0.99	—
Height (cm)	145.29 ± 12.83	143.88 ± 10.45	160.00 ± 8.67
Weight (kg)	40.96 ± 11.83	38.76 ± 10.26	63.08 ± 10.12
Waist (cm)	65.99 ± 9.80	63.67 ± 8.89	82.67 ± 8.79
Hip (cm)	80.62 ± 10.25	79.28 ± 8.10	93.65 ± 5.93
Waist/Hip	0.82 ± 0.06	0.80 ± 0.06	0.88 ± 0.08
Fat mass (kg)	9.97 ± 6.39	8.74 ± 5.43	17.17 ± 5.42^a^
SBP (mmHg)	112.54 ± 17.19	106.52 ± 11.21	122.23 ± 18.89
DBP (mmHg)	64.32 ± 8.90	67.00 ± 8.32	80.60 ± 11.79
HDL (mg/dl)	58.93 ± 12.34	58.52 ± 11.54	44.66 ± 10.10
TC (mg/dl)	170.11 ± 28.15	169.73 ± 26.74	191.55 ± 36.02
TG (mg/dl)	76.43 ± 43.69	81.17 ± 51.08	162.44 ± 104.53
FPG (mmol/l)	4.64 ± 0.42	4.75 ± 0.56	4.62 ± 0.49
Insulin^b^ (μU/mL)	9.66 ± 6.53	8.98 ± 7.24	7.50 ± 4.57
HOMA-IR^b^	2.02 ± 1.45	1.95 ± 1.76	0.83 ± 0.50

^a^Fat mass (KARE n = 7013).

^b^Insulin and HOMA-IR (KoCAS-1 n = 380, KoCAS-2 n = 1200, KARE n = 7495); Body mass index, BMI; High-density lipoprotein cholesterol, HDL; Total cholesterol, TC; Triglycerides, TG; Fasting plasma glucose, FPG.

**Table 2:** Association statistics for the rs10879834 SNP (Risk/Other allele, T/C).

	KoCAS-1		KoCAS-2		KARE	
	beta ± SE	*P*	beta ± SE	*P*	beta ± SE	*P*
Risk allele frequency	0.713		0.726		0.709	
BMI	0.340 ± 0.063	1.81E-07	0.075 ± 0.037	3.94E-02	0.036 ± 0.017	3.30E-02
WAIST	0.247 ± 0.062	8.46E-05	0.088 ± 0.036	1.41E-02	0.005 ± 0.017	7.53E-01
WT	0.204 ± 0.052	9.90E-05	0.062 ± 0.029	3.30E-02	0.031 ± 0.017	6.47E-02
HIP	0.244 ± 0.056	1.74E-05	0.078 ± 0.032	1.38E-02	0.017 ± 0.016	2.86E-01
HDL	−0.025 ± 0.0143	1.02E-01	0.245 ± 0.448	5.85E-01	0.676 ± 0.174	6.97E-01
CHOL	1.194 ± 1.913	5.33E-01	0.406 ± 1.047	6.99E-01	1.199 ± 0.613	5.03E-02
TG	0.066 ± 0.036	6.85E-02	0.081 ± 1.967	9.67E-01	−0.356 ± 1.803	8.43E-01
FPG	0.068 ± 0.029	3.69E-02	1.067 ± 1.067	5.99E-02	−0.004 ± 0.009	6.75E-01
INS0	−0.410 ± 0.507	4.18E-01	−0.151 ± 0.310	6.26E-01	−0.297 ± 0.493	5.48E-01
HOMA-IR	0.079 ± 0.113	4.89E-01	−0.055 ± 0.065	4.01E-01	−0.005 ± 0.009	5.52E-01

All traits were tested by multivariate linear regression analysis in an additive genetic model (1-d.f.) after adjustment for age, sex, and recruitment area as covariates. Non-diabetic individuals were tested for FPG in the KARE study. Body mass index, BMI; High-density lipoprotein cholesterol, HDL; Total cholesterol, TC; Triglycerides, TG; Fasting plasma glucose, FPG.

**Table 3:** DNA methylation analysis in T2D-discordant monozygotic twins (n = 12 pairs).

ID	Location	Map info	Chr	Gene	giDMR_P	DMR_P
cg27154343	5′UTR	75603480	12	KCNC2	0.196	0.011

Genetically independent DMRs (giDMRs) represent pure environmental effects and p-values were obtained from one-sample parametric t-test. DMRs were calculated using a linear mixed effects model with random effect (family structure) and fixed (age, sex, and BMI) effects.
