# Supplementary material for: An integrative study identifies KCNC2 as a novel predisposing factor for childhood obesity and the risk of diabetes in the Korean population
Source: Sci Rep. 2016 Sep 14;6:33043. doi: 10.1038/srep33043 (PMC5022012; doi:10.1038/srep33043)

An integrative study identifies *KCNC2* as a novel predisposing factor for childhood obesity and the risk of diabetes in the Korean population

Joo-Yeon Hwang[1†], Hyo Jung Lee[2†], Min Jin Go[1], Han Byul Jang[2], Sang Ick Park[2], Bong-Jo Kim[1¶], Hye-Ja Lee[2¶]

Supplementary Table 1. Association statistics for lead SNPs in the stage 1.

**CHR**	**POSITION**	**SNP**	**Candidate Gene**	**Risk allele**	**Other allele**	**beta ± SE**	***P***
12	rs10879834	75152254	KCNC2	T	C	0.340 ± 0.063	1.81E-07
11	rs2512887	131466218	NTM	G	A	0.310 ± 0.067	4.20E-06
12	rs10505912	24042275	SOX5	C	T	0.261 ± 0.058	9.41E-06

Supplementary Table 2. Summary statistics of T2D-discordant monozygotic twins (12 pairs)

	T2D	Normal
Sex (M/F)	6/6	6/6
Age (yr)	42.83 ± 8.28	42.83 ± 8.28
Body mass index (kg/m^2)	24.79 ± 3.47	25.14 ± 3.28
Fasting plasma glucose (mmol/L)	8.15 ± 2.49	6.55 ± 1.37

Supplementary Figure 1. Mahattan plot

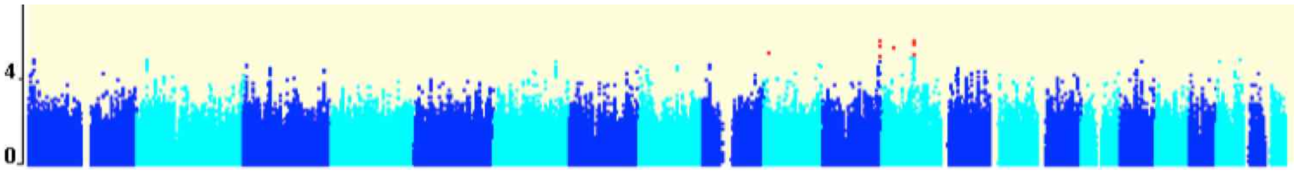

Supplementary Figure 2. Quantile-Quantile plot

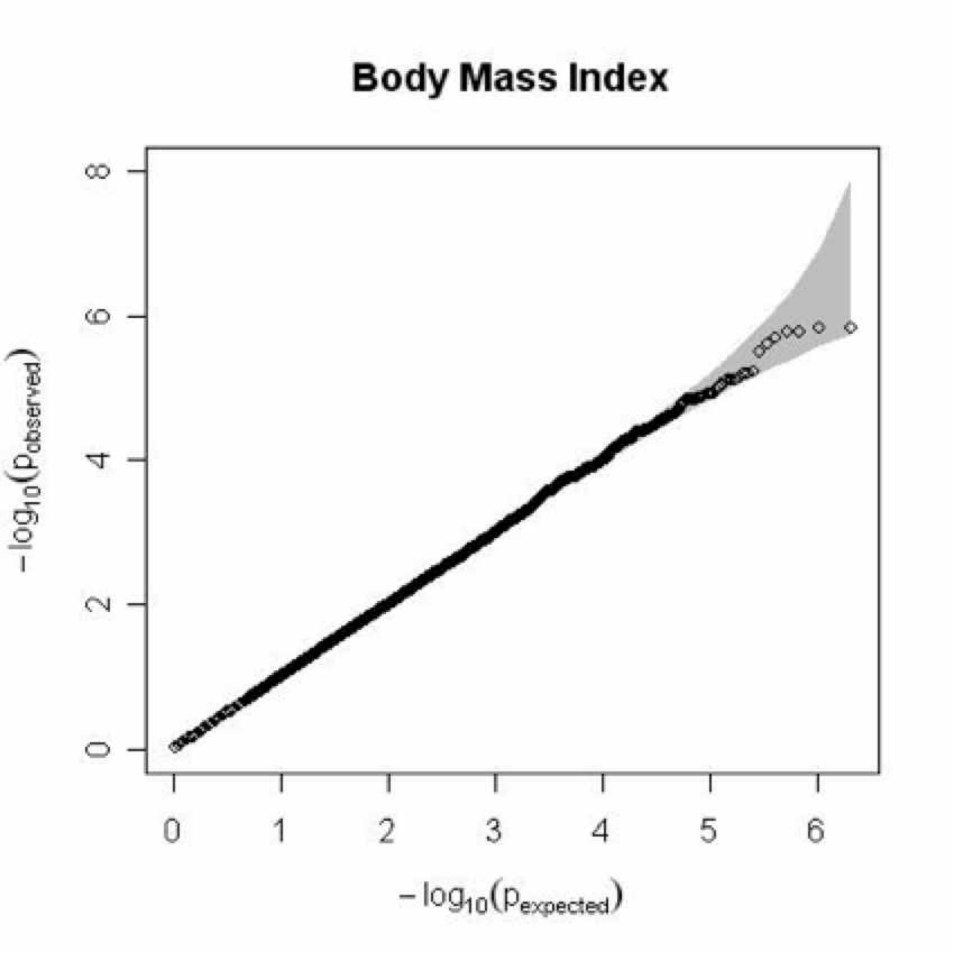

Supplementary Figure 3. *cis-me*QTL plot using the MuTHER resource

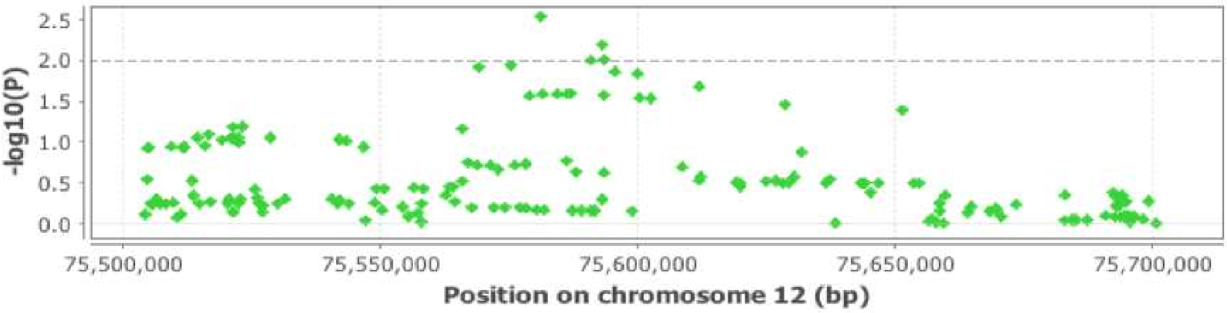

The *cis*-meQTL associations were calculated by Spearman's rack correlation tests. MuTHER (Multiple Tissue Human Expression Resource) data was tested by *cis-me*QTL analyses using adipose tissue data from a population of 428 female twins (856 individuals).

Supplementary Figure 4. Gene expression signatures in T2D cases (blood) from GEO database resource

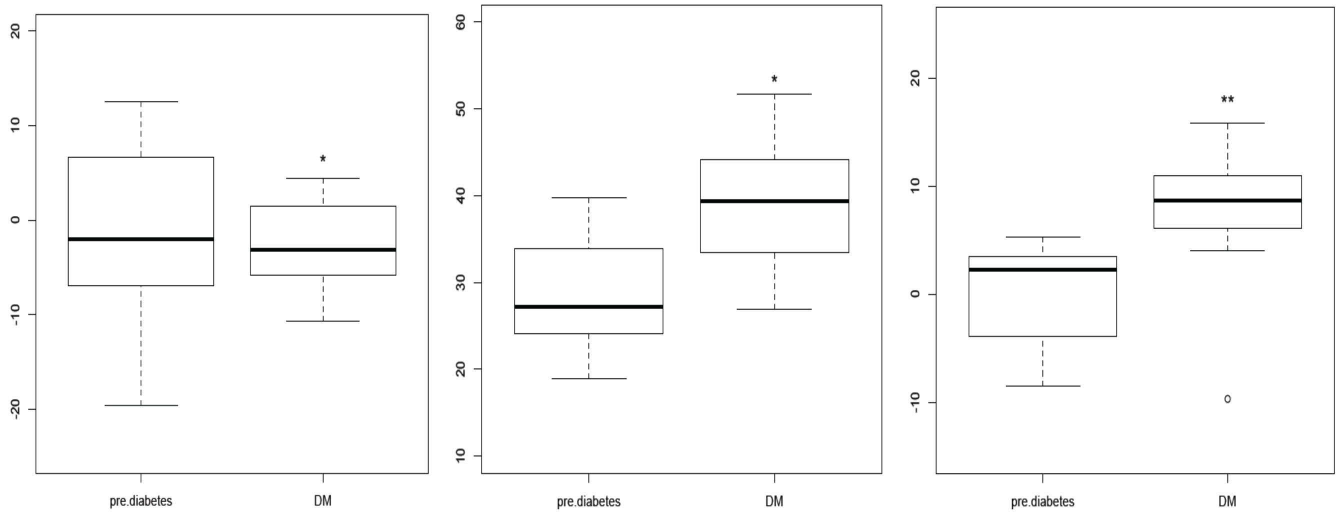

KCNC2 (p = 3.46e-02) *PRKAR2A* (p = 2.34e-02) *PRKAR2B* (p = 2.86e-03)

Gene expression data set (GSE26168/GPL6883) was retrieved from the NCBI Gene Expression Omnibus (GEO)

Supplementary Figure 5. Middle-length blots and two exposures of Figure 6

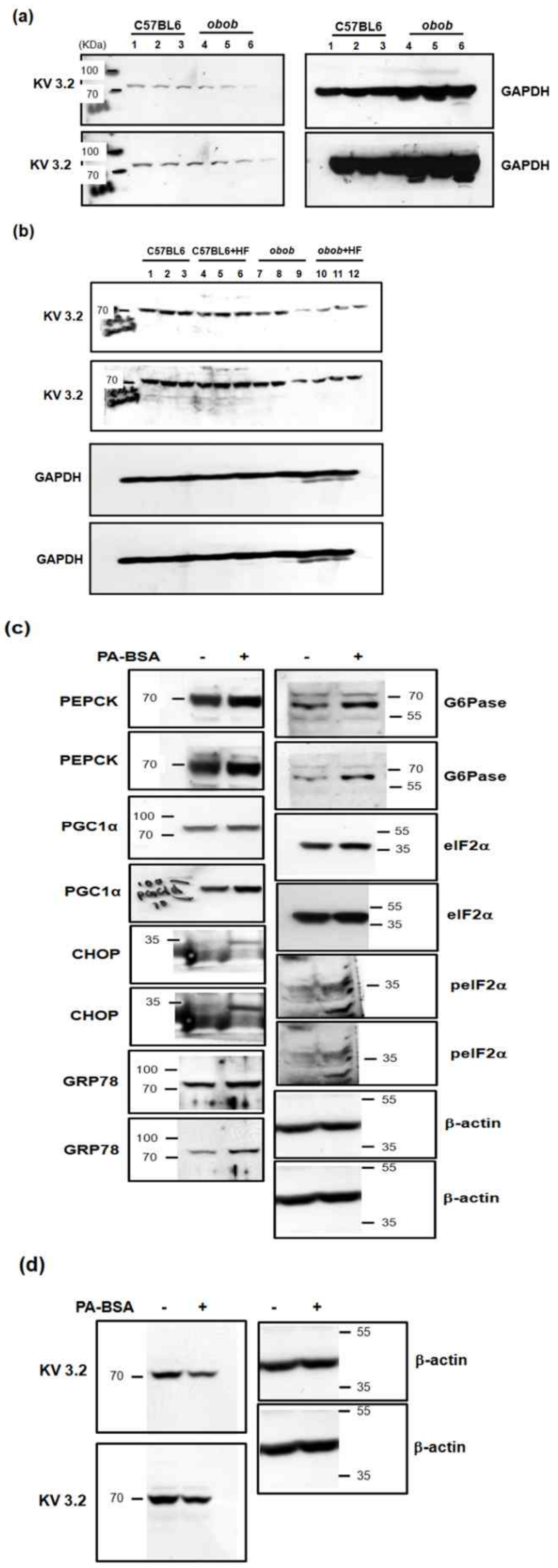

Supplementary Figure 6. Middle-length blots and two exposures of Figure 7

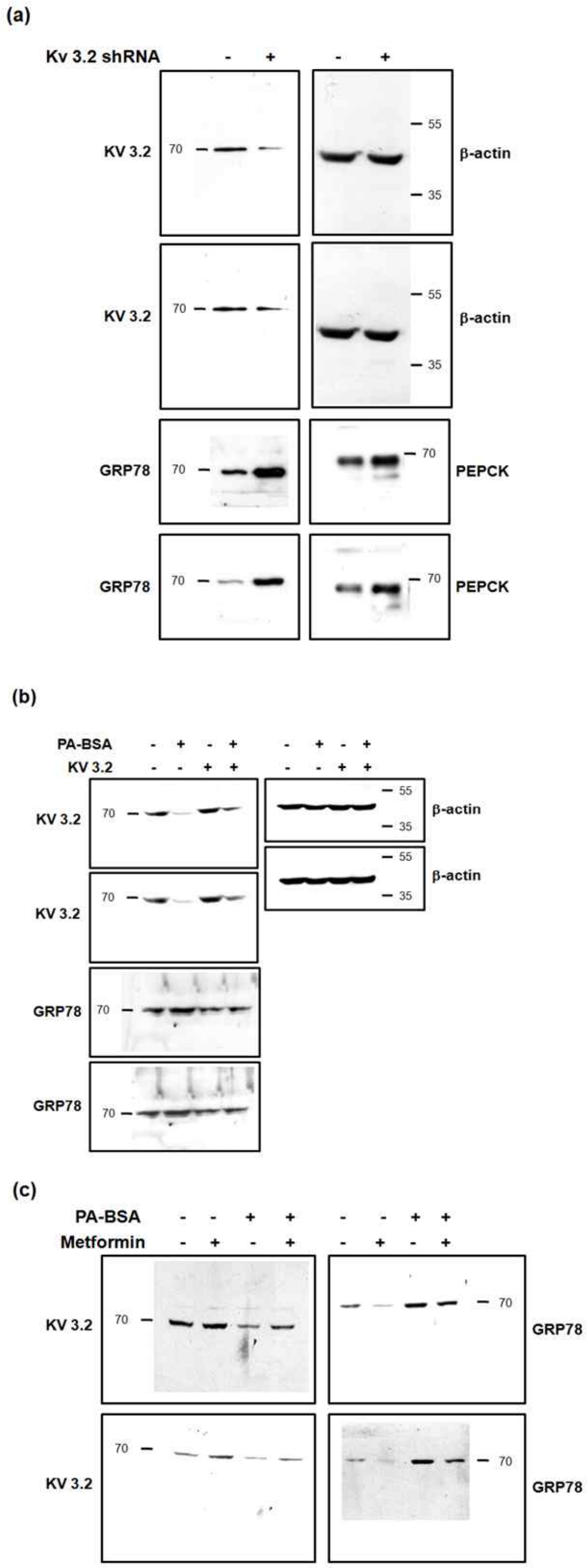

Supplement: Supplementary Information [file srep33043-s1.pdf]
